# Surgical outcome of atypical subtrochanteric and femoral fracture related to bisphosphonates use in osteoporotic patients with or without teriparatide treatment

**DOI:** 10.1186/s12891-017-1878-5

**Published:** 2017-12-13

**Authors:** Wen-Ling Yeh, Chun-Yi Su, Chia-Wei Chang, Chien-Hao Chen, Tsai-Sheng Fu, Lih-Huei Chen, Tung-Yi Lin

**Affiliations:** 1 Department of Orthopedic Surgery, Division of Trauma, Chang Gung Memorial Hospital, Linkou branch, Bone and Joint Research Center and Chang Gung University, Taoyuan, Taiwan; 2 Department of Orthopaedic Surgery, Chang Gung Memorial Hospital, Keelung branch, Bone and Joint Research Center, and Chang Gung University,, Taiwan F7, No 222 Mai-King Road, Keelung, Taiwan

**Keywords:** Atypical femur fractures, Bisphosphonates, Osteoporosis, Teriparatide

## Abstract

**Background:**

Atypical subtrochanteric fracture and femoral fracture have been considered to be rare complications related to long-term bisphosphonates use. A reduced bone turnover rate may lead to delayed bone healing. Limited data have revealed that teriparatide treatment may reverse the effect of bisphosphonates and be effective in bone healing.

**Methods:**

We reviewed patients with atypical subtrochanteric and femoral fracture related to bisphosphonates use between January 2008 and December 2014. Thirteen female patients were enrolled. Radiographic findings were compatible with the characteristics of atypical fracture. Surgical intervention was performed for all, and teriparatide use was advised postoperatively. Outcome measures included perioperative results, and clinical and radiographic outcome.

**Results:**

Of the 13 female patients enrolled, 10 had subtrochanteric and 6 had proximal femoral fracture; 3 had bilateral fractures. The mean age of the patients at surgery was 70.15±6.36 years. Most fractures (68.8%) presented prodromal thigh pain. All patients were treated with an intramedullary fixation system without severe complications. The patients were divided into 2 groups based on whether they had received treatment with teriparatide or not. The mean time to bone union was 4.4 months in the teriparatide-treated group, and 6.2 months in the non-teriparatide-treated group (*p*=0.116). Six patients (75%) in the teriparatide-treated group and 4 (50%) in the non-teriparatide-treated group (p= 0.3) achieved bone union within 6 months. The means of the modified Harris Hip Score and Numerical Rating Scale were significantly better in the teriparatide-treated group at postoperative 6 months. Seven patients had the same ability to walk at the 1-year follow-up as they did before the atypical fracture.

**Conclusions:**

Teriparatide treatment in patients with atypical fracture may help in fracture healing, hip function recovery, and pain relief in this reduced bone turnover patient group.

## Background

Bisphosphonates are anti-resorptive agents approved by the FDA, and are a 1st-line osteoporosis treatment. Their use may increase bone mineral density and effectively prevent osteoporotic-related fracture [1, 2]. However, bisphosphonates may cause certain kinds of adverse events. Atypical subtrochanteric fracture and femoral fracture are considered to be complications with a low incidence that are related to long-term bisphosphonates use. The clinical symptoms and signs include a prodrome of thigh pain, minor trauma or even no trauma, and some radiographic characteristics [3–5].

Atypical fracture may be associated with prolonged suppression of bone remodeling caused by bisphosphonates use [6–8]. Radiographic characteristics of atypical fracture are transverse to oblique fracture with a thickening lateral cortex and spiking of the medial cortex, usually localized from the subtrochanteric region to the proximal femur [5, 9]. In addition, the reduced bone turnover rate resulting from bisphosphonate use may lead to delayed bone healing at the fracture site in osteoporotic patients [10, 11]. In an effort to resolve this issue, anabolics may be used to reverse low bone turnover and stimulate new bone formation. Teriparatide, a recombinant of human parathyroid hormone, is considered an anti-osteoporotic agent that is potent in new bone formation and improves bone healing in patients with nonunion or delayed nonunion [12, 13]. Teriparatide treatment was found to refresh bone remodeling after bisphosphonates use and possibly be effective in promoting bone healing [14, 15], although the definite mechanism has not been elucidated. Several case reports [16–20] and series studies [21, 22] have mentioned the benefits of teriparatide for patients with atypical fracture. Nevertheless, more data on the treatment outcome of atypical fracture is urgently needed to clarify this issue. The purpose of our study is to present the surgical outcome of atypical subtrochanteric and femoral fracture related to bisphosphonates use in patients with or without teriparatide treatment.

## Methods

**Fig. 1:**
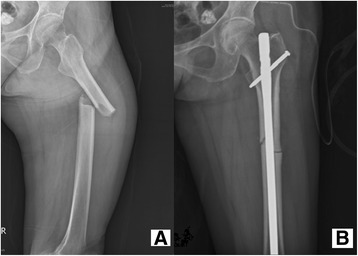
A patient suffered from left thigh pain after a minor trauma. **a** Left proximal femur fracture with short oblique fracture line, lateral cortical thickening and medial cortical spike. **b** After reduction, internal fixation with intramedullary nail

We retrospectively reviewed patients with the diagnosis of atypical subtrochanteric and femoral fracture related to bisphosphonates use between January 2008 and December 2014. We recorded patient information, including clinical manifestations, medication history, radiographic characteristics, operative methods, treatment outcome and follow-up results. Thirteen female patients were enrolled in the study and had noticed thigh pain after minor trauma. Radiographic findings were compatible with the characteristics of atypical fracture, including transverse or short oblique fracture without bony comminution (Fig. 1). Lateral cortical beaking and medial spiking were also noticed.

For all these patients, bisphosphonates use was ceased after atypical fracture was diagnosed, but calcium supply was continued. Surgical intervention was performed for all patients via internal fixation with an intramedullary device. Teriparatide, at a once-daily 20μg dose, was advised postoperatively for continuity in the treatment of osteoporosis and better bone healing.

### Outcome measures

#### Perioperative assessment

Clinical assessment was performed based on occurrences of perioperative complications and their causes, including wound problems, anesthesia-related morbidity, or even mortality.

#### Radiographic outcome

Radiological assessment was based on follow-up radiographs taken postoperatively and at 1, 2, 3, 6, and 9 months and 1 year post-operation. Fracture union, delayed union and implant material failure were analyzed by 2 independent observers using radiographic images, with both anteroposterior and lateral views. According to the United States Food and Drug Administration (US FDA), nonunion is defined as a fractured bone that has not completely healed within 9 months of injury and that has not shown progression of healing on serial radiographs for 3 consecutive months [23]. Delayed union was defined as the lack of bone union evidence at postoperative 6 months.

#### Functional outcomes

Functional assessments by phone were retrospectively made by independent reviewers using the modified Harris Hip Score (HHS) at 3 months, 6 months, and 1 year post-operation, and the Numerical Rating Scale (NRS) pain score at the postoperative follow-up, and at 3, 6, and 9 months, and 1 year post-operation. Walking ability was also assessed at the 1-year postoperative follow-up, and the patients were placed into 1 of 4 groups based on their walking ability: walking without aid, walking with a cane, walking with a walker, and non-walking.

The modified HHS is a functional assessment scale for the hip with a maximum score of 100 points; its measures include pain, mobility, daily activities and range of motion [24]. The NRS pain score graded from 0 to 10 points was used for pain evaluation. The scale reflects subjectively proportional pain levels ranging from 0 (no pain) to 10 (worst possible pain), as reported by the patients.

#### Statistical analysis

Statistical analysis was performed with SPSS, using the Mann-Whitney U test and Fisher’s exact test. Differences were defined as statistically significant when *P* < 0.0.

## Results

Thirteen female patients were enrolled: 10 had subtrochanteric and 6 had proximal femoral fractures; 3 had bilateral fractures. All patients had a medical history of exposure to bisphosphonates – alendronate, for at least 2.5 years (2.5 to 6 years). The demographic data of the study population is summarized in Table 1. The mean age of the patients at surgery was 70.15 years (range, 58 to 79 years). Eleven of the 16 atypical fractures presented prodromal thigh pain (68.8%). The implant choices of the 10 patients with subtrochanteric fracture included intramedullary nailing for 2 patients, recon nail for 5, Gamma nail (Gamma3, Stryker) for 2, and Proximal Femoral Nail Antirotation (PFNA, Synthes) for 1 patient. The 6 patients with proximal femur fracture all underwent intramedullary nailing fixation. There were no major complications for these patients. Two patients (12.5%) were found to have poor wound healing in the perioperative period, but no infection was evident. There was no deep vein thrombosis, no other anesthesia-related complications and no mortality in this series.

**Table 1:** Demographic and clinical characteristics of the 2 patient groups

	All	Teriparatide-treated	Non-Teriparatide-treated	*p*-value
Atypical fracture	16	8	8	
Age (yr)	70.15	70.25(68)	69.25(72.5)	0.916
Duration of Bisphosphonate use (yr)	4.04	4.36 (5)	4 (4)	0.696
Proximal femur fracture	6	3	3	1
Subtrochanteric fracture	10	5	5	1
Time to union (month)	5.3	4.4 (4)	6.2 (5.6)	0.093
Union at 6 months (%)	10 (62.5%)	6 (75%)	4 (50%)	0.608
Implant failure	1	0	1	1

Data presented as mean (median)

At the follow-up radiographic assessment, 10 atypical fractures (62.5%) presented good bone union within 6 months. Five fractures (31.25%) were recognized as delayed union in the radiographic assessment, but they had all achieved bone union and good consolidation at the 9-month follow-up. The other patient presented implant failure and nonunion. Six patients agreed to receive teriparatide treatment initially for at least 6 months. The patients with atypical femur fractures were divided into 2 groups based on whether they had received teriparatide treatment or not. One patient refused teriparatide in the beginning. However, she began using teriparatide when delayed union of the 1st atypical femur fracture was found; the contralateral femur fracture occurred soon after. There were 8 atypical fractures in the teriparatide-treated group and 8 in the non-teriparatide-treated group (Table 1). The mean time to bone union was 4.4 months (range, 2.3 to 8.2 months) in the teriparatide-treated group, and 6.2 months (range, 4 to 9 months) in non-teriparatide-treated group (p=0.093). Six patients (75%) achieved bone union within 6 months in the teriparatide-treated group and 4 (50%) in the non-teriparatide-treated group. There was no time-to-union data for the patient with nonunion and implant failure.

In terms of functional outcome, the mean of the modified HHS was 79 in the teriparatide-treated group and 68.29 in the non-teriparatide-treated group at postoperative 6 months, revealing a significant difference. (p=0.021) The NRS at postoperative 6 months showed a mean of 2.63 in the teriparatide-treated group, and 3.88 in non-teriparatide-treated group, which was a significant difference (p value = 0.042). Before fracture, 6 patients (46.2%) could walk without aid, 5 (38.5%) could walk with the aid of a cane, and 2 (15.4%) needed a walker when walking. Evaluation of walking ability at the 1-year postoperative follow-up revealed 7 patients (53.8%) had the same ability to walk as before the atypical fracture (Table 2).

**Table 2:** Functional outcome of the 2 patient groups

Modified Harris Hip Score	All	Teriparatide-treated	Non-Teriparatide- treated	*p*-value
Postoperative 3 months	67.14	72 (70)	62.29 (65)	0.114
Postoperative 6 months	73.64	79 (78)	68.29 (73)	0.021*
Postoperative 1 year	81.57	84.29 (88)	78.86 (83)	0.384
Numerical Rating Scale				
Postoperative	7.38	7.25 (7)	7.5 (7.5)	0.582
Postoperative 3 months	3.94	3.75 (3.5)	4.13 (4)	0.413
Postoperative 6 months	3.25	2.63 (3)	3.88 (4)	0.042*
Postoperative 9 months	1.94	1.38 (1.5)	2.5 (3)	0.139
Postoperative 1 year	1.13	1 (1)	1.25(1)	0.628
Recovery to pre-OP walking ability (%)	7 (53.8%)	5 (71.4%)	2 (33.3%)	0.217

*p value < 0.05

Data presented as mean (median)

**Fig. 2:**
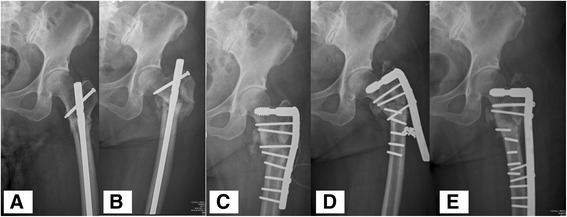
Another patient, who had a hypertension, diabetes mellitus, and breast cancer history, received bisphosphonates for 5 years. Left thigh pain developed after a minor trauma, and left atypical subtrochanteric fracture was noted. **a** Internal fixation with intramedullary nail was performed. **b** The reduction was failure and implants back out at post-operative one year due to nonunion. **c** Revision surgery was then performed with a Dynamic Condylar Screw with allobone grafting. **d** Left thigh pain 4 months later with difficulty in weight-bearing. Radiography showed implant failure again with multiple broken screws. **e** Another revision surgery was performed with a longer Dynamic Condylar Screw with allobone grafting. The patient was under follow-up in the outpatient department

One patient suffered from fracture nonunion and implant failure (Fig. 2): this 61-year-old female, who had a history of hypertension, diabetes mellitus, and breast cancer, received bisphosphonates for 5 years. Left thigh pain developed after minor trauma, and left atypical subtrochanteric fracture was diagnosed by radiography. Internal fixation with intramedullary nail was performed at first, but the reduction failed and the implant backed out due to nonunion at 1-year post-operation. Two revision surgeries were performed as a result of nonunion and implant failure. The patient was followed up at the outpatient department.

## Discussion

In our series, 13 female patients with 16 fractures were treated via internal fixation with intramedullary osteosynthesis. The teriparatide-treated group showed better results with a significantly higher modified HHS and lower NRS at 6 months post-operation, compared with the non-teriparatide-treated group. The benefit of teriparatide use may be due to the reverse effect it has on suppression of bone remodeling caused by prolonged bisphosphonate use.

Atypical subtrochanteric and femoral fracture after prolonged bisphosphonate use for osteoporosis is considered to be a stress fracture after minor or even no trauma. The mean duration of bisphosphonate treatment was usually more than 4 years, but has differed in different studies [10, 25, 26]. In this study, all patients were female and had had alendronate treatment for a mean of 4.04 years (ranging from 2.5 to 6 years). The exact pathogenesis of atypical fracture has not been well proven, although some reports have proposed a possible mechanism [27, 28]. From the current data, we may conclude that the effect of decreased bone remodeling may also lead to reduced crack removal [29, 30], which may result in a high delayed union rate or even nonunion [31, 32]. In our study, most patients (75%) with teriparatide treatment could achieve bone union within 6 months, with a mean of 4.4 months. Although the p value was not significantly different between the 2 groups, teriparatide treatment may improve bone healing and union for patients with atypical fracture.

Several studies have mentioned the benefit of teriparatide treatment for patients with atypical fracture [16–22]. The cited references are summarized in Table 3. Nevertheless, because of the low incidence of atypical fracture, there is a lack of randomized controlled trials and large cohort studies that mention surgical outcome and the role of teriparatide. Chiang et al. [21] reported a case series of atypical femoral fractures treated with teriparatide. Five patients received teriparatide 20μg daily, which resulted in a 2-3-fold increase in bone remodeling markers and fracture healing. The 9 patients with no teriparatide treatment suffered from poor fracture healing with ongoing pain. In 2014, Shane et al. [33], in the 2nd report of the Task Force of the American Society for Bone and Mineral Research (ASBMR), reviewed a limited amount of high-evidence data, and recommended the discontinuation of bisphosphonate. Instead, they recommended giving a calcium and vitamin D supplement and considering the use of teriparatide for patients who appear not to heal with conservative therapy.

**Table 3:** Summary of the cited references on treating atypical fracture with teriparatide

Reference	Case No.	Sex	Bisphosphonates exposure	Surgery or not	Duration of teriparitide	Result summary
Huang et al. 2012 [16]	1	F	3 yr	No	9 mo	After 1 mo, pain and tenderness improved After 9 mo, symptoms disappeared After 15 mo, fracture line completely healed
Gomberg et al. 2011 [17]	1 (bilateral)	F	13 yr	No	21 mo	After 6 mo, decreased edema at the fracture sites with cortical bridging on MRI. After 16 mo, pain-free and return to work After 21 mo, complete healing on MRI.
Carvalho et al. 2011 [18]	1	F	4 yr	IM nail	3 mo	After 1 mo, closure of the right femur fracture After 3 mo, sβ-CTX and serum osteocalcin increased by 22 and 300%, respectively
Tsuchie et al. 2015 [19]	2 (1 bilateral)	Both F	4/6 yr	Locking plate	12 mo	After 3 and 6 mo, fracture line almost healed. After 3 and 2 yr, no thigh pain, respectively.
Mastaglia et al. 2016 [20]	1	F	7+ yr	IM nail	3 mo	After 1 mo, significant pain relief and walk without support After 3 mo, fracture healed on CT
Chiang et al. 2013 [21]	14	13F, 1M	4-10 yr	3 patients	6 mo	Five treated with teriparatide, 2-3 fold increase in bone remodeling markers and fracture healing. Of 9 patients without teriparatide, 3 had prophylactic surgery. All remaining 6 had nonunion.
Saleh et al. 2012 [22]	10 (4 bilateral)	All F	10±5 yr	IM nail in 7	8 patients for 24 mo	Five fractures without radiolucent fracture line treated conservatively with protection and teriparatide. Of 9 fractures with a radiolucent fracture line, 2 were successfully treated with teriparatide. Six treated by surgery and teriparatide for 3 mo. One treated only by surgery.

IM, intramedullary, yr, year; mo, month; sβ-CTX, serum β-carboxyterminal telopeptide; MRI, magnetic resonance imaging; CT, computed tomography

Ekström et al. [34], in a prospective cohort study, reported that half of their patients with subtrochanteric fracture could not recover to their pre-fracture level of activities of daily living, and they had a high mortality rate, up to 25%, at the 2-year follow-up. However, there was very limited data to evaluate the treatment strategy for atypical fracture and there was no strong conclusion. In our series, 10 atypical fractures (62.5%) presented good bone union within 6 months, 5 fractures (31.25%) were recognized as delayed union, and 1 patient had nonunion with implant failure. Two revision surgeries with bone grafting were performed, and the patient is still undergoing evaluation and follow-up at the outpatient department. Seven patients (53.8%) in our series had the same ability to walk as before the atypical fracture. Teo et al. [35] recorded the surgical outcome of 33 consecutive female patients with atypical subtrochanteric fracture. Delayed fracture union was noted, with a mean period of 10 months for radiological union. Seven patients (23%) suffered implant failure, and the authors concluded that atypical subtrochanteric fracture is associated with slow healing and prolonged postoperative immobility. The higher implant failure rate in that study than in our series may be explained by the more common use (69.7%) of extramedullary fixation. The implant failure rates in Teo’s study were 29% and 11%, for extramedullary versus intramedullary devices, respectively. Lovy et al. [36] recently reported 11 patients with atypical femur fracture that received bone marrow aspirate concentrate after intramedullary nail fixation. Results showed that the treated patients had a significantly decreased time-to-union than the control group. All patients in this group achieved bone union within 1 year.

Even though atypical fractures have become a concern with bisphosphonate use, osteoporosis-related complications such as hip fracture may indeed cause consequent morbidity and mortality. Bisphosphonates use has been well reported to decrease the coincidence of hip fracture by 30% [37–39]. Edward et al. [40] reported the occurrence of bisphosphonate-associated non-healing femoral fractures in a review of data from the US FDA Adverse Event Reporting System (FAERS) (from 1996 to 2011), and concluded that the benefits of bisphosphonates use are 100-fold greater than the risk of atypical femoral fractures.

There are several limitations in this case series study. The small sample size and retrospective study design may result in any comparison between the 2 treatment groups having less strength. Also, teriparatide treatment was recommended to every patient, but the patient and family made the final decision, which could result in bias. However, the rarity of atypical femur fractures may lead to difficulty in designing a prospective randomized controlled study. The inclusion of only females is another limitation. Because of the developmental course of bisphosphonates use and their use as a treatment for postmenopausal osteoporosis, female patients may receive bisphosphonates several years earlier than male patients, which will result in a more prolonged exposure among female patients. This may explain the rarity of atypical femur fracture among male patients. Therefore, further evaluation and investigation, including larger patient numbers, are necessary.

## Conclusions

Teriparatide treatment for patients with atypical fracture may help in fracture healing, hip function recovery, and pain relief in this reduced bone turnover patient group. The bone union rate may also be improved with teriparatide use, although the data was not statistically significant.

## Abbreviations

FDA: US Food and Drug Administration
HHS: Harris Hip Score
NRS: Numerical Rating Scale
ASBMR: American Society for Bone and Mineral Research
FAERS: FDA Adverse Event Reporting System
